# "There's no place like home" A pilot study of perspectives of international health and social care professionals working in the UK

**DOI:** 10.1186/1743-8462-2-25

**Published:** 2005-10-27

**Authors:** Anna Moran, Susan Nancarrow, Allister Butler

**Affiliations:** 1 Trent RDSU, Institute of General Practice and Primary Care, School of Health and Related Research, The University of Sheffield, ICOSS Building, 219 Portobello, S1 4DP, Sheffield, UK; 2 Faculty of health, Canterbury Christ Church University College, Kent, UK

## Abstract

**Background:**

Many countries are reporting health workforce shortages across a range of professions at a time of relatively high workforce mobility. Utilising the global market to supply shortage health skills is now a common recruitment strategy in many developed countries. At the same time a number of countries report a 'brain drain' resulting from professional people leaving home to work overseas. Many health and social care professionals make their way to the UK from other countries. This pilot study utilises a novel 'e-survey' approach to explore the motives, experiences and perspectives of non-UK health and social care professionals who were working or had worked in the UK. The study aims to understand the contributions of international health and social care workers to the UK and their 'home' countries. The purpose of the pilot study is also in part to test the appropriateness of this methodology for undertaking a wider study.

**Results:**

A 24-item questionnaire with open-ended and multiple choice questions was circulated via email to 10 contacts who were from a country outside the UK, had trained outside the UK and had email access. These contacts were requested to forward the email to other contacts who met these criteria (and so on). The email was circulated over a one month pilot period to 34 contacts. Responses were from physiotherapists (n = 11), speech therapists (n = 4), social workers (n = 10), an occupational therapist (n = 1), podiatrists (n = 5), and others (n = 3). Participants were from Australia (n = 20), South Africa (n = 10), New Zealand (n = 3) and the Republic of Ireland (n = 1). Motives for relocating to the UK included travel, money and career opportunities. Participants identified a number of advantages and disadvantages of working in the UK compared to working in their home country health system. Respondents generally reported that by working in the UK, they had accumulated skills and knowledge that would allow them to contribute more to their profession and health system on their return home.

**Conclusion:**

This pilot study highlights a range of issues and future research questions for international learning and comparison for the health and social care professions as a result of international workforce mobility. The study also highlights the usefulness of an e-survey technique for capturing information from a geographically diverse and mobile group of professionals.

## Background

World over, countries use strategies to manage health workforce shortages such as improving retention, attracting 'non-traditional' entrants and attracting back 'returnees' [1]. International recruitment is another popular solution to overcoming shortfalls of health providers [2-6]. Recruitment strategies aside, health workforce mobility is increasing, particularly the flow of health and social care professionals to and from the UK.

### Health and social care professionals in the UK

Most of the available data surrounding health worker migration relates to the nursing and medical professions. For instance, in the UK and Ireland there were more overseas additions to the in 2000/2001 UK nursing register than home country registrants [1] and two thirds of new registrants to the UK General Medical Council in 2003 were from overseas, mostly from outside the European Economic Area [7]. Less research has been undertaken into the mobility of the allied health and social care workforce.

During the 1990s, registration of non-UK trained physiotherapists ranged from 26 to 42 percent; sourced mainly from Australia, South Africa and New Zealand [8]. The Health Professions Council (HPC), the regulatory body responsible for registration of allied health professionals and clinical scientists in the UK, captures data on applications for registration by profession and country of origin. In the 2004 / 05 financial year there were 3,515 international registrations. Nearly one third of these (n = 1,339) were physiotherapists, followed by radiographers (n = 681), occupational therapists (n = 668) and biomedical scientists (n = 363). These reflect the relative proportions of the total registrations for the respective professions. The major donor 'continents' during the same period were Europe (excluding the UK) (n = 129), Africa (n = 110), Asia (n = 107), followed by Oceania (n = 105) (which includes Australia and New Zealand) [Source: HPC 2005].

### Australian and New Zealand diaspora

The Australian and New Zealand diasporas have similar histories. It is estimated that one million (out of twenty million) Australians are overseas at any one time [9] and the primary destination of resident professionals leaving Australia and New Zealand on a permanent or long-term basis is the United Kingdom [9-11]. In addition, recent changes to international immigration laws for skilled professionals has made professional migration more seamless [9,11].

### 'Brain drain'

Not surprisingly, the concept of 'brain drain' resulting from professionals leaving their country of residence to work overseas has created great concern for 'source' or 'donor' countries, who also report shortages of health and social care professionals [9,12-15]. Brain drain is deemed particularly pernicious in developing nations, particularly African countries, and compounded by the fact that emigrating skilled workers are more likely to stay in their host country [6]. It is well documented that recruitment of health personnel from developing countries threatens the operation of crucial health programs in these countries[3].

### 'Brain circulation'

In contrast, recent research indicates international migration of skilled workers is often temporary and the mobility of this workforce generates global benefits by improving knowledge flows and satisfying the demand for skills, often termed 'brain circulation' [9,11,13]. However to date there is little research specifically analysing the benefits of skilled migration in health and social care.

### Push and Pull factors

It has been suggested that push and pull factors motivate workers to leave one country and seek employment in another [8,16] where *push *factors are motives to leave home countries such as low pay, limited career opportunities, unemployment or civil unrest and *pull *factors are motivations or conditions that attract migrants to other countries such as demand for workers or a higher standard of living. This idea has been further developed to categorise workers as permanent or temporary movers based on their motives for leaving their home country [16]. For example permanent movers may be 'economic migrants' who are attracted to better standards of living and who may send money to their home country or 'career migrants' who are attracted to enhanced career opportunities. Temporary movers include those on a 'working holiday' where expertise is used to finance travel, or 'the study tour' where new knowledge and techniques are acquired for use when they return home.

Westcott and Whitcombe [17] suggest that the benefits offered by the globalisation of occupational therapy have not been fully realised, particularly in reference to education. One study has been conducted to inform writers of UK speech and language therapy curricula by utilising the perspectives of current and past international students [18].

The motivations, experiences and perspectives of international health and social care professionals however have not been thoroughly explored, particularly in reference to workforce dynamics, workforce flexibility [19-23] and the understanding and management of the global flow of health and social care workers. This pilot study aims to capture preliminary data on the perspectives and motives of health and social care professionals of non-UK origin and training who have at some stage moved to and worked in the UK.

## Methods

The pilot study used a novel approach to data collection to capture a range of views from health and social care workers on their experiences of working in the UK. The data collection relied on the assumption that many foreign trained workers living in the UK have access to email and that many international workers have wide international networks.

As a result, we piloted the use of an 'e-survey' which was distributed by the researchers via email to 10 contacts inviting them to participate on the basis that they had worked in the UK at some stage. The participants represented a range of disciplines, having worked in different settings (hospital, community etc) and different geographic locations across the UK. The first round participants were accessed through personal and professional networks. Each of those contacts was asked to forward the e-survey to their international contacts and so on creating a snowball sampling effect. We allowed a four week time frame for replies. This method was chosen over other more traditional survey methods as it was a quick and inexpensive way to gain preliminary insight into an internationally diverse and geographically varied group of health and social care workers. The purpose of the pilot study was, in part, to test the appropriateness of this methodology for undertaking a wider study.

Inclusion criteria were health and social care professionals from any country other than the UK, who trained outside the UK and who have previously worked or are currently working in the UK. UK nationals and UK trained health and social care professionals were excluded. The intention of the research was to capture information from this pool of relatively mobile international health and social care staff.

The 24 item questionnaire included closed and open-ended questions and was designed to capture demographic information about the participants, their profession, perceptions of training, career development opportunities and learning experiences. It was piloted with four expatriate allied health professionals resulting in the deletion of two questions.

The survey was initially circulated as a Microsoft Word attachment. However feedback from some participants indicated that it was not always possible to download or open the attachment, so the survey was then embedded into the body of the email. The researchers established a web-site on which detailed information about the research was available, including the protocol and details of the researchers and from which further questionnaires could be downloaded. All but two surveys were returned electronically. These were faxed and posted to the researchers.

All data were entered into a Microsoft Excel spreadsheet. Numeric data have been presented in descriptive numerical form. Where large amounts of qualitative data were received, they have been summarised for this paper. For the purpose of this paper, the phrase 'country of origin' refers to the nationality of the professional.

## Results

### Response

Ten e-surveys were emailed in the first round and responses were received from 34 participants within the one month pilot, of which one was incomplete. The profile of the respondents is summarised in Table 1.

**Table 1:** Profile of Respondents

		**N**	**%**
**Responses**	Complete	33	
	Incomplete	1	
			
**Profession**	Physiotherapy	11	32
	Social Work	10	29
	Podiatry	5	15
	Speech and Language Therapy	4	12
	Occupational Therapy	1	3
	Nurse	1	3
	Medical Doctor	1	3
	Medical Transcript Editor	1	3
			
**Age**	Mean 31 (25–55), Median 28		
			
**Country of Origin**	Australia	20	60
	South Africa	10	30
	New Zealand	3	
	Rep of Ireland	1	
			
**Number of years qualified**	Mean 7 (2–19), Median 4.5		

Few respondents reported difficulties obtaining a UK visa, the majority using a working holiday visa (available to Commonwealth citizens aged 17–30) or work permit. Professionals had worked in a median number of 2 cities (range 1–15, mean 3) and usually as a 'locum' where locum work is defined as temporary or contractual employment. The median time spent in the UK was 3 years (range 3 months – 8 years, mean 2.5 years). Employment was typically gained through a recruitment agency in the UK or in their home country. There were 27 professionals still in the UK, most of whom were working in Social Services (n = 9), for the NHS full time (n = 8) or as a locum (n = 7) or in private practice (n = 6). Over half of the respondents (67%, n = 23) reported they would not stay in the UK permanently. Table 2 summarises these results.

**Table 2:** Work details of respondents

		**N**	**%**
**Number of locations worked**	Mean 3 (1–15), Median 2		
	One location	14	
	Two locations	6	
	Three locations	5	
	> Three locations	9	
			
**Main location in UK**	London	9	26
	Essex	6	17
	Sheffield	3	8
	Oxford	3	8
	Other (Edinburgh, Worcester, Cambridge, Nottingham, Clacton-on-sea, Cardiff, Gosport, Loughton, Halifax, West Grinstead, Rotherham, Falkirk)		
			
**Visa difficulties**	Yes	6	18
	No	28	82
			
Visa Type *	Working Holiday	13	
	Work Permit / sponsorship	11	
	Ancestry	6	
	British Passport	4	
	Highly skilled migrant programme	1	
	Other	2	
			
**Means of securing job in UK**^+^	Agency in home country	10	
	Locum Agency	6	
	Agency in the UK	15	
	Advertisement in the home country	4	
	Advertisement in the UK	6	
	Word of mouth	7	
	Other	1	
			
**Why work in the UK^$^**	Travel	29	
	Money	23	
	Career	16	
	Partner	3	
	Other	4	
			
**Areas worked in UK^$^**	Locum	21	
	NHS	15	
	Social Services	10	
	Private practice	7	
	Research	5	
	Self Employed	4	
	Teaching	2	
			
**Current area of work in UK^$^**	Locum	7	
	NHS	8	
	Social Services	9	
	Private	6	
	Research	4	
	Self Employed	2	
	Teaching	1	
	N/A	5	
	Incomplete	1	

* May have held more than one visa

^+^May have held more than one job

^**$ **^Multi-answer question

### Motivation to work in the UK

Respondents were asked why they initially chose to work in the UK. Travel (n= 29), money (n = 23) and career opportunities (n = 16) were the primary motives expressed. One respondent answered ...*to experience living in a country other than my home country.' (Physiotherapist, Australia)*

### Expectations prior to working in the UK

Respondents were asked what their expectations of working in the UK were prior to their arrival and how they compared to their experience. There were mixed responses, many reporting they believed the UK would be superior to their home country in terms of resources, professional expertise and funding (n = 5) ; others assumed it would be the same (n = 15).

Thought it would be similar to Australia – maybe not quite so advanced with their techniques. Experience was pretty much what I thought it would be – depends on the different hospitals, which is the same back home. (Speech and language therapist, Australia)

I thought that the workforce would be more superior and be able to provide good guidance. I expected there to be far more resources to enable service users to achieve an element of self actualization. Experience: poor management, poor team and case planning, lack of resources and especially money to provide for the needs of service users. (Podiatrist, Australia)

I just expected to work and earn money to travel but in reality it is a time when you can really work on your professional development, which is what I am doing. (Physiotherapist, Australia)

### Skills and Training

Respondents were asked what skills or training could have better equipped them to work in the UK and to describe their perceptions of the quality of training in the UK. Many felt they had adequate skills and training to work in the UK (n = 15) but highlighted greater knowledge of the health and social care systems would have been beneficial particularly those working in social services (n = 6).

My training and skills were of English standards. The only adjustments I had to develop skills around was to know the culture of the community and adjust strategies of intervention. (Speech and language therapist, Australia)

Most perceived that the undergraduate training in the UK was of a lower standard than their country of origin (n = 23) but that opportunities for continuing professional education were superior in the UK (n = 10). Some felt that their undergraduate training equipped them more thoroughly to enter the workforce with more confidence in their role than their UK counterparts.

I believe the broad 4 year undergraduate training in Australia is of very high quality. This allows for multi-skilling and confidence from day one. Therapists trained in Australia tend to be a lot more confident in their skills and are more used to working within multidisciplinary teams. (Occupational therapist, Australia)

Others felt that each system had its advantages.

The UK training is more practice based and reflective. SA training is more theory based. (Social worker, South Africa)

UK pre-qualifying training inferior due to shorter course length. Good first year graduate program compensates for this. (Physiotherapist, New Zealand)

### Attractiveness of working in the UK

Respondents were asked what was good about working in the UK. The most attractive features were greater access to Continuing Professional Development (CPD), wider variety of specialisation, more career opportunities and a well-defined career structure. Experiencing a different system and culture was also a theme as well as travel and greater earning power.

Accessibility to latest research and professional development (remote location of many Australian practices limits this). Number of jobs available with very acute caseload (far fewer in Aust). Opportunity to develop quickly as a therapist given our undergraduate skills and general confidence as therapists. Close proximity to Europe and travel opportunities. (Speech and language therapist, Australia)

### Difficulties working in the UK

Respondents were asked what was not good about working in the UK. The most unappealing features included large waiting lists and correspondingly large caseloads, poor recognition or respect as a professional, the bureaucracy, the weather, or for some professional groups, racism.

Understaffing of all health professions; Too much paperwork and repetition of paperwork; Reduced hospital standards; Distance from home. (Podiatrist, Australia)

Some people find it difficult to accept that although not trained in the UK the level of skill you bring into the profession is of high value. (Social worker, South Africa)

Participants were also asked how the status of their profession in the UK compared to their country of origin. The majority (n = 24) felt the status of their profession was lower in the UK than in their home country compared to eight who felt it was the same.

I expected to retain the same high status that my profession has back home with other professionals and the communities who appreciate the services provided by the profession. This was completely opposite when I got here (UK) and shocking to me. This discrepancy creates difficulties in working with partners to bring about desired change. (Social worker, South Africa)

### Benefits and suggestions for country of origin

When asked how their country of origin could benefit or learn from their experience of working in the UK, most responded they had gained a much broader skill base and knowledge of how a different system works.

I have such a vast array of experiences now to draw on, both good and bad which I can take home with me. I think I am much more worldly now. (Physiotherapist, New Zealand)

I have experienced many management styles, and government agendas, and would be able to take the advantages and disadvantages of these systems back to Aust. and formulate better solutions to problems. (Speech and language therapist, Australia)

Due to excellent continuing education [in the UK] I feel I will have a more up to date knowledge base which I will attempt to pass on when I begin working again at home. (Physiotherapist, Australia)

## Discussion

### Method

The e-survey technique was chosen over other more traditional survey techniques as it is a quick and cost-effective way to gain preliminary insight into a geographically and demographically diverse group of professionals. The mobility of the diasporas makes them a particularly difficult group to access in a systematic way and, as this study has demonstrated, health and social care workers may work in a number of different cities during their time away. Additionally the e-survey was deemed the most appropriate method given that this project was proposed as a pilot study with the intention to trial the methodology to identify pertinent themes for future research, rather than generate statistically generalisable findings.

The e-survey technique provided an opportunity to capture a global 'before and after' perspective for many professionals who were now living and working at 'home', in a new city or another new country. These professionals may have proved more difficult to sample using a professional register such as the Health Professions Council which captures 'inflow' registration information only[8]. In order that a larger, more systematic e-survey be repeated, electronic networks for diasporas which exist in some countries [24] could be accessed. Alternatively, professional newsletters and journals may facilitate a more targeted approach to specific disciplines or groups.

The limitations of the e-survey approach include the inability to follow-up non-respondents, as once the initial round of surveys had been circulated the researchers had no control over the distribution network. For the same reason, no response rate can be calculated as the denominator is unknown.

Additionally, it was difficult to avoid the potential sources of bias inherent in this type of study. For instance, by circulating the survey electronically, we could only access those workers who use email, and there was no way of knowing how the IT literacy of the respondents impacted on the response rate. The selection of the initial ten respondents may also have introduced bias, however the researchers reiterate this intention of this pilot study was to identify future research themes.

### Push and Pull factors

The primary motivations for this group of health and social care professionals to work in the UK were travel, money and career opportunities. These motivations fit with research conducted by Buchan [16], whereby most respondents in this study were 'temporary movers', pursuing 'the working holiday'. This was particularly true for those from Australia and New Zealand. Equally, respondents were relatively young. These findings correspond to research conducted by Birrell et al [9] who reports 70% of Australian professionals who work overseas return and are usually aged between 20 and 30. Also demonstrated in this study was a trend for internationally trained health workers to fill temporary, locum positions in the NHS. A report for the UK Chartered Society of Physiotherapists [8] recognises a key 'pull' factor for overseas physiotherapists is the relative ease with which they can find comparatively well paid temporary work in the UK, giving them greater choice over the location and duration of employment. These findings are supported by Allan and Larsen [26] for international nurses and O'Hagan [26] for Australian medical radiation graduates. The results also reinforce the perception that South Africans are more likely to be 'permanent movers', a trend recognised by Cerventes [6].

### Perceptions and Experiences

When examining the perceptions and experiences of the respondents, it is important to remember their original motivations to move to the UK and how this may affect their experiences and perceptions. For example the majority of respondents were motivated to move to the UK by the opportunity to travel. Perceptions may then be from the perspective of a holiday maker, working to fund travels rather than from the perspective of a full time employee working to pay a mortgage, for example. Motives can also change over time depending on different circumstances, for example the motives of and incentives for migrating nurses to the UK have been shown to change over time as personal and socio-economic conditions alter [25]. Respondents' experience of their 'home' health system and organisational culture would also significantly contribute to the forming perceptions and opinions about the UK health organisation and culture. Additionally respondents have experienced a mixture of 'British' culture and NHS organisational culture which together have influenced their perceptions and opinions.

#### Resources

The qualitative responses demonstrate themes of dissatisfaction and discontent with NHS bureaucracy and lack of resources. This has also been reported in the UK with claims that people leave the UK public sector primarily due to bureaucracy and paper work, lack of resources, lack of autonomy and feeling undervalued[27]. Similarly, a cohort of UK physiotherapy students and professionals perceived physiotherapy in the NHS to have high levels of stress and workload, staff shortages and poor equipment [28].

#### Undergraduate skills and training

There was a clear perception that undergraduate training is comparatively better outside the UK. This may in part be explained by a discrepancy in the length of training undertaken. South African trained social workers have traditionally had a longer programme of training at undergraduate level, and their education has been granted the status of full degree for longer than their UK equivalents [29]. This is also true for Australian allied health and social work undergraduate degrees, many of which are 4 year qualifications compared to the 3 year UK equivalent [30]. As these professionals have not directly experienced training in the UK, these findings need to be interpreted cautiously. Given these perceptions, it is not surprising respondents felt their skills and training gained at 'home' adequately equipped them to undertake work in the UK health and social care sectors. Further research comparing these perceptions to UK trained professionals working in Australia, South Africa or New Zealand would add value to these findings.

#### Continuing Professional Development

Many of the respondents reported on the 'value adding' attributes of practicing in the UK. These included the extensive opportunity for post qualifying training or Continuing Professional Development (CPD), a more defined and progressive career structure and greater availability of specialisation. One study confirms these perceptions, reporting that UK physiotherapists and prospective UK physiotherapy students perceive that the career structure in the NHS and variety in work are desirable qualities of NHS physiotherapy as a career [28].

In many cases local health authorities in the UK offer financial contributions towards tuition fees as well as protected time to pursue both academic coursework and CPD. The growth in and support for CPD in Britain is in part due to explicit Department of health NHS policies and frameworks which outlined the need for delivery of high quality care and clinical excellence in the NHS [31,32]. Although Australian professional bodies are equally as attentive to CPD [30], improved access to CPD in the UK may also be explained by the size of the British health and social care system proportionally providing greater numbers of courses for a larger health workforce. Further research is needed to explore this possibility. A smaller and more convenient geographic area for accessing CPD in the UK compared to remote, rural areas of Australia or South Africa may also be a contributing factor. South Africa however has not yet developed a coherent curriculum that focuses on CPD for social work graduates [29].

#### Professional Status

Undergraduate educational differences may also speak of the issue of professional identity and the reported contrast between professional status of health and social care work in the UK and other countries. Turner [33] compared the status of physiotherapy in Australia to the UK finding Australian general public and physiotherapy students perceive physiotherapy to have a higher occupational prestige than their UK equivalents. The health and social care professionals who responded to our study indicated that they entered the UK system from a very different professional perspective, accounting in part for the difference in perceived professional image. Another study [28] of UK physiotherapy students and professionals, showed that they perceive the general public and other health care professionals to have a lack of recognition for physiotherapy. Negative perceptions of professional status have also been noted within podiatry in the UK [34-37], the USA [39] and, to some extent, Australia [36]. An interesting discrepancy therefore emerges, whereby the perception of lower professional status in the UK is reported along side the perception of improved pay and career opportunities.

### International information sharing

This pilot study has highlighted the extent to which international information sharing and collaboration can benefit both 'home' and the UK, particularly in reference to service development, education and career development. The extent to which this mutual learning is realised is dependent on how accessible international registration is [23]; how compatible training and education are; the degree to which health and social care communities embrace the knowledge and expertise foreign workers offer their country and the wisdom returning professionals bring home.

### Implications for future research

Many themes have emerged from this study that give rise to further research questions. Of particular interest is the relationship between status, pay and career opportunities in the UK and other countries; the effects of different undergraduate training on accessibility and quality of a 'global' health workforce; and the effect of culture on health and social care systems. Further studies utilising a larger sample size may aid in exploring these themes.

## Conclusion

This pilot study demonstrates that international health and social care professionals who have worked in the UK have accumulated vast amounts of experience and knowledge. The study captured the perspectives and experiences of a group of professionals who have gained experience working in different health systems and cultures. This combined with a high percentage of those professionals returning to their country of origin, makes for a new class of highly resourceful and skilled professionals, with global ideas and resources to share with colleagues. It would be valuable to further pursue in depth what this growing group of internationally skilled health and social care professionals offer both their country of origin and the UK. As one respondent commented:

*It's a bit like doing rotations to different departments but to different countries*.

## Competing interests

The author(s) declare that they have no competing interests.

## Authors' contributions

AM and SN contributed jointly to the research design and data collection. Both authors were involved in the analysis, interpretation of data and preparation of the final manuscript. AB was consulted about the design of the research, provided literature, access to networks of clinicians and South African perspective.
